# Validity of a booklet to promote the health of people with diabetes in the face of COVID-19

**DOI:** 10.1590/0034-7167-2022-0472

**Published:** 2023-05-08

**Authors:** Carla Lidiane Jácome dos Santos, Alex dos Santos Silva, Waleska de Brito Nunes, Jacira dos Santos Oliveira, Cizone Maria Carneiro Acioly, Thalys Maynnard Costa Ferreira, Marta Miriam Lopes Costa, Lidiane Lima de Andrade

**Affiliations:** I Universidade Federal da Paraíba. João Pessoa, Paraíba, Brazil; II Universidade Federal de Campina Grande. Cuité, Paraíba, Brazil; III Centro Universitário de João Pessoa. João Pessoa, Paraíba, Brazil

**Keywords:** Diabetes *Mellitus*, Coronavirus Infections, Health Promotion, Educational Technology, Validation Study

## Abstract

**Objectives:**

to validate the content and appearance of a booklet to promote the health of people with diabetes *mellitus* in the face of COVID-19.

**Methods:**

a methodological study, carried out in a virtual environment with experts who had practical and scientific experience in diabetes *mellitus*, from November 2021 to February 2022. Data were analyzed using the Content Validity Ratio.

**Results:**

twenty-seven experts from different states of Brazil and with different academic backgrounds participated in the study. In content validity, reviews were suggested in items in relation to objectives and structure, in appearance validity, adjustments in layout were suggested.

**Conclusions:**

the results showed that the booklet achieved adequate content and appearance validity rates. Therefore, when adapting to experts’ suggestions, it becomes an accurate tool.

## INTRODUCTION

The new coronavirus pandemic (COVID-19) has caused intense economic and health instability, as well as changes in people’s lifestyle, especially those who are part of the most vulnerable groups, such as people living with diabetes *mellitus* (DM), which has been a challenge in the context of health care and nursing^(1-2)^.

DM is an endocrine-metabolic disease resulting from the lack and/or inability of insulin to control blood glucose, resulting in hyperglycemia. Hyperglycemia causes increased release of advanced glycation end products (AGEs), release of pro-inflammatory cytokines, and oxidative stress. This deregulation of glucose metabolism, followed by the release of enzymes related to tissue damage, causes people with DM to have a deregulated immune system, causing excessive inflammatory responses, which make them more prone to infections with worse outcomes^(3)^.

Evidence indicate that people with DM and COVID-19 have a higher risk of admission to the Intensive Care Unit (ICU) and a longer hospital stay, with severe complications of the disease and higher mortality, when compared to people who do not have DM^(4-5)^. Furthermore, during the COVID-19 pandemic, people with DM had their routines changed, as social distancing limited self-care practices, such as performing physical activities, purchasing antidiabetic drugs and glucose test strips, in addition to food care. These conditions were identified as factors that led this group to present worsening in glycemic control^(6-7)^.

Thus, considering the changes in lifestyle imposed by the COVID-19 pandemic, it is essential to promote health promotion actions in order to avoid as much as possible the exposure of this group to Severe Acute Respiratory Syndrome Coronavirus 2 (SARS-CoV-2), plus measures that culminate in an effective line of care, in order to achieve glycemic control, the reduction of risks of complications and mortality among those who may contract COVID-19, making them active in the self-care process^(2,8-9)^.

Although it is surrounded by a great paradigmatic complexity, with regard to its concept, health promotion is understood as the set of actions that encourage individual and collective autonomy, leading subjects to know and control the determining factors of their health. Health promotion works with the idea of multiple responsibility for problems and solutions^(10)^.

In this sense, educational technologies have been shown to facilitate the health promotion process, bringing knowledge and encouraging adherence and improvements in self-care, contributing to a better quality of life for people. They can be used as support in the teaching-learning process, when they present current, attractive information with scientific evidence^(11)^. Therefore, recognizing the incipience of studies in this area and the contribution of the technology proposed here, for the scientific community, for assistance and for people with DM, it is essential that this be submitted to an assessment.

## OBJECTIVES

To validate the content and appearance of a booklet to promote the health of people with DM in the face of COVID-19.

## METHODS

### Ethical aspects

The study was guided by Resolution 466/12 of the Brazilian National Health Council, which deals with the Guidelines and Regulatory Norms for Research with Human Beings in Brazil. Received approval by the Research Ethics Committee of the *Hospital Universitário Alcides Carneiro* at the *Universidade Federal de Campina Grande*. Moreover, the principles governed by Circular Letter 02/2021 were considered, which presents guidelines for procedures in research with any stage in a virtual environment.

### Study design, period, and place

This is methodological research for validity of an educational technology (booklet). Initially, the booklet was elaborated, through an integrative review, which aimed to map scientific evidence on health promotion measures to improve the quality of life of people living with DM during the COVID-19 pandemic. The final sample consisted of 17 articles, which listed individual, collective and governmental health promotion measures. After defining the booklet content, meetings were held with professionals specialized in design, layout and illustration, together with the researchers, preparing the first version of the booklet.

Booklet content and appearance validity with experts took place from November 2021 to February 2022.

### Population or sample; inclusion and exclusion criteria

To compose the panel of experts, with regard to selection criteria, practical and scientific experience were considered, considering that these are fundamental elements for a satisfactory level of expertise^(12-13)^. Thus, health professionals involved in the care of people with DM, with at least one year of experience and/or professionals who have a *lato sensu* graduate degree in the area of DM or a *stricto sensu* graduate degree, having developed a dissertation or thesis in the area of DM, were included. Those who did not respond to the invitation letter within two weeks of receipt and those who agreed to respond, but did not assess after three attempts of the established deadline were excluded.

For the sample calculation, the formula for infinite population was used, whose n= Z^2^_1-α/2_.P(1-P)/e^2^, where Z_1-α/2_ refers to the confidence level adopted; P represents the expected proportion of experts, indicating the adequacy of each item; and “e” represents the acceptable proportion difference in relation to what would be expected^(13)^. Adopting a minimum expected proportion of 80%, a confidence level of 95% and an acceptable error of 15%, a sample of 27 experts was obtained.

### Data collection and organization

To identify experts, a search was carried out on the *Plataforma Lattes*, on the Brazilian National Council for Scientific and Technological Development (CNPq - *Conselho Nacional de Desenvolvimento Científico e Tecnológico*) website, using the “resume search” tool in “advanced search”, using the following specifiers: DM (subject search), applying the academic training and professional performance filters, according to selection criteria. The snowball technique was also used, in order to assist in recruitment, consulting the relational universe of experts, who recommended other professionals with the same attributes.

After the search and identification, experts were contacted through an invitation letter (via email), in which the research objectives and the importance of their contribution to the validity process were explained. The experts who agreed to participate in the study received the Informed Consent Form via Google Forms, the colored booklet in a preliminary version and the validity instruments, containing characterization data and criteria for validating the booklet content and appearance, in addition to allowing the addition of suggestions and comments on the material.

The instrument used for content validity considered 18 items, subdivided into the following aspects: objectives (purposes, goals or purposes); structure/presentation (organization, structure, strategy, coherence and sufficiency); and relevance (significance, impact, motivation and interest)^(14)^. With regard to appearance validity, 10 questions related to illustration and layout were used^(15)^. The answers to all questions were presented on a three-point scale, where 0=disagree, 1=partially agree and 2=completely agree.

### Data analysis

Therefore, for each item on the form, the degree of agreement among experts was calculated using the Content Validity Ratio (CVR), which assesses content based on agreement among experts regarding how much a given item can be “essential to the test”, “useful to the test, but not essential” or “not necessary”^(16)^. It should be noted that the CVR is a strong psychometric indicator, a tool capable of providing the best validity assessment^(17)^.

The formula for calculating the CVR was CVR = (Ne-N/2)/(N/2), where Ne corresponded to the number of experts who indicated the option “totally agree”, and N, to the total number of experts. Thus, item CVR(I-CVR) was calculated, as well as aspect CVR (A-CVR), demonstrating the validity based on the average values of I-CVR^(16)^.

In addition to this, the CVR was used in order to reduce the risk of bias related to the size of the expert panel, since the critical value of the CVR depends on the number of experts included^(16)^. Therefore, as the number of experts who participated in the study was 27, the critical value or cut-off point of the CVR used was 0.407. Therefore, items with a CVR equal to or greater than this point were considered to have good evidence of content and appearance validity^(18)^. Items with a lower CVR were reassessed in the booklet, according to experts’ suggestions.

## RESULTS

The panel was composed of 27 experts, aged between 24 and 52 years, with a mean age of 35.7 (±8.1) years. The time taken to conclude the undergraduate course ranged from 3 to 29 years, with an average of 12.6 (±6.9), in which 15 (55.6%) completed it more than 10 years ago. Furthermore, 25 (93%) of participants claimed to have experience in caring for people with DM, ranging from zero to 19 years, with an average of 7.7 (±6.0) years, according to data in Table 1.

**Table 1:** Sociodemographic and professional characterization of experts, João Pessoa, Paraíba, Brazil, 2022

Variáveis	n	%
Sex		
Female	25	93.0
Male	02	7.0
Age		
24-36	16	59.3
37-52	11	40.7
State where they live		
Paraíba	10	37.0
Ceará	09	33.0
São Paulo	03	11.0
Pernambuco	02	7.0
Rio Grande do Norte	01	4.0
Minas Gerais	01	4.0
Santa Catarina	01	4.0
Undergraduate course		
Nursing	19	70.0
Nutrition	05	19.0
Physical therapy	01	4.0
Pharmacy	01	4.0
Graduation course completion time		
0 to 10 years	12	44.4
>10 years	15	55.6
Master’s degree		
Yes	21	78.0
No	06	22.0
Doctoral degree		
Yes	10	37.0
No	17	63.0
Experience in caring for people with diabetes mellitus		
Yes	25	93.0
No	02	7.0
Experience time		
0 to 10 years	19	70.0
>10 years	08	30.0

In content validity, two items did not obtain a minimum CVR of 0.407 in item adequacy to the teaching-learning process and clarification of doubts about the topic addressed, with CVR of 0.259 and 0.111, respectively (Table 2). They were reassessed and changed, according to experts’ suggestions.

**Table 2:** Content validity of an educational technology by experts in the field of diabetes *mellitus*, João Pessoa, Paraíba, Brazil, 2022

Aspects	Items	I-CVR^*^	A-CVR ^†^
Objectives	1. Includes proposed theme	0.556	0.407
	2. Adequate for the teaching-learning process	0.259	
	3. Clarifies doubts about the topic addressed	0.111	
	4. Provides reflection on the topic	0.556	
	5. Encourages behavior change	0.556	
Estrutura	6. Appropriate language for the target audience	0.333	0.430
	7. Appropriate language for educational material	0.481	
	8. Interactive language, allowing active involvement in the educational process	0.333	
	9. Correct information	0.037	
	10. Objective information	0.407	
	11. Clarifying information	0.333	
	12. Necessary information	0.407	
	13. Logical sequence of ideas	0.481	
	14. Current topic	1.000	
	15. Appropriate text size	0.481	
Relevance	16. Encourages learning	0.852	0.778
	17. Contributes to knowledge in the area	0.852	
	18. Arouses interest in the topic	0.630	

* *Item Content Validity Reason; †Aspect Content Validity Ratio.*

In the aspect related to booklet structure and relevance, experts considered the information objective and necessary, with a logical sequence of ideas, current theme and adequate text size, reviews were suggested in items that questioned whether language was appropriate for the target audience, whether language was interactive and allowed active involvement in the educational process, both with CVR= 0.333, correct information and clarifying information, with CVR of 0.037 and 0.333, respectively (Table 2).

In appearance validity, the booklet was judged positively by experts, with changes being suggested only in the layout aspect, in the text formatting item, in terms of size, the latter with a CVR of 0.111 (Table 3).

**Table 3:** Appearance validity of an educational technology by experts in the field of diabetes *mellitus*, João Pessoa, Paraíba, Brazil, 2022

Aspects	Items	I-CVR^*^	A-CVR ^†^
Illustration	1. Are the illustrations necessary to understand the content?	0.926	0.719
	2. Do illustrations motivate manipulation of printed material?	0.926	
	3. Do the illustrations elucidate the content?	0.481	
	4. Is the number of illustrations adequate for the material content?	0.556	
	5. Do the illustrations have lines and/or resolution adequate for the target audience?	0.704	
Layout	6. Are the text formatting in terms of font (type) and letter size adequate?	0.111	0.556
	7. Is the visual composition attractive and organized?	0.556	
	8. Is the choice of colors adequate?	0.704	
	9. Is the page size adequate?	0.778	
	10. Is the number of pages adequate?	0.630	

* *Item Content Validity Reason; †Aspect Content Validity Ratio.*

Chart 1 presents a summary of changes that were suggested by experts for each instrument domain that was sent via Google Forms. To expose the suggestions, items that had a CVR lower than 0.407 were taken into account.

**Chart 1:** Summary of suggestions from experts in the area of diabetes *mellitus* regarding content and appearance of an educational technology, João Pessoa, Paraíba, Brazil, 2022

Aspects	Experts’ suggestions
Objectives: booklet’s purposes, goals or purposes	*Adequate to the teaching-learning process:* focus on information that addresses the DM topic, relating it to information about COVID-19 in general; explain foreign terms.
	*Clarifies doubts about the topic addressed:* specify which foods are considered healthy and do not increase blood glucose level and which are considered unhealthy and harmful to blood glucose control; add strategies for performing physical exercises; include the general recommendation of the amount of water; add anguish, discouragement and sadness to the anxiety part; add how much social distancing should be.
Structure/presentation: organization, structure, strategy, coherence and sufficiency	*Appropriate language for the target audience:* opt for more explanatory and less scientific language.
	*Interactive language, allowing active involvement in the educational process:* summarize contents, including more images. Long texts make reading more tiring.
	*Correct information:* correct the figure on the cover in which the woman has her hand on top of a mask; when recommending hand hygiene, preference is given to liquid soap, regardless of whether it is neutral or not; correct the page that talks about masks, removing the valve from the N95 mask; correct the information that surgical masks protect against aerosols; correct the information on blood glucose, according to the current guideline of the Brazilian Society of Diabetes (2021-2022).
	*Clarifying information:* add a page talking about the correct way to put on and take off masks; reinforce the rational use of masks.
Layout	*Text formatting in terms of font (type) and font size are adequate:* standardize the font size, as on some pages it is too small, as in the second part of the title.

## DISCUSSION

The production of educational material is the best way to summarize, standardize and formalize various guidelines regarding user care with health promotion, in addition to providing subsidies for self-management and providing knowledge to make it possible to change lifestyle habits^(19-21)^. Moreover, the content and appearance validity process by experts made many contributions to the material, since aspects related to content and graphic presentation were adjusted, bringing it closer to the reality experienced by people with DM in the face of COVID-19.

Validity with experts from different academic backgrounds and from different regions of Brazil gave educational technology the combination of different knowledge and different cultures, standardizing guidelines. This allows for the formation of materials with better content and better ways of presentation, and this process is essential to develop a booklet^(22)^.

Professionals from the areas of nursing, nutrition, physiotherapy and pharmacy participated in this study. Other studies have already reported the importance of multidisciplinary participation in the validity process, showing that appreciation by professionals from different areas brings greater quality to the material and an improvement through critical-reflective thinking^(15)^. Consequently, it provides a more comprehensive care, considering that information from specific areas is assessed by people who dominate the knowledge of their profession^(19-20,23)^.

However, unlike this study, the literature presents the validity of educational materials with the participation of only expert nurses^(24)^. Nursing stands out, with its greater representation and experience in the development and validity of educational technologies^(25)^.

Regarding the results of content validity, it can be seen that the CVR of aspects related to objectives, structure and relevance presents relevant and accurate content, reaching acceptable levels of good content validity. For experts, the technology content provided reflection on the subject and encouraged a change in the behavior of people living with DM. Confirming this finding, a methodological research to validate an educational booklet content for asthma control and management in children also showed acceptable values^(26)^.

However, in relation to the CVR of each item, it was observed that the items Adequate to the teaching-learning process, Clarifies doubts about the topic addressed, Appropriate language for the target audience, Interactive language, allowing active involvement in the educational process, Correct information and Clarifying information was presented below the cut-off point. Adjustments related to content improvement were suggested by experts, including text spelling adequacy, text reformulation, modification of the sequence of pages to facilitate the understanding of content and change of terms or expressions, making reading simpler, to favor the maximum possible interpretation by the target audience.

In order to improve content, all experts’ suggestions were accepted, taking into account that, for an educational technology to be adequate to the profile of its target audience, it must, in addition to assertive and fundamental information, present itself as attractive and didactic, with regard to the language used, since mistaken and incomplete information can mislead the target audience or impair the understanding of the transmitted information.

It is quite common changes in validity of educational technologies, according to experts’ suggestions, in an attempt to make content clearer, more complete and more usual for the target audience^(27)^.

With regard to appearance validity, the CVR of the item related to text formatting in terms of font and letter size reached a value below the recommended value. Experts proposed that font sizes were standardized, maintaining uniformity between titles, topics and texts; thus, there were changes in the font size to facilitate reading, making content more attractive.

Illustrations and layout are essential elements that produce an attractiveness. The importance of these resources favors an aesthetically well-presented and stimulating material, making reading more pleasurable^(28)^. According to the suggested changes, the booklet went through a process of re-elaboration, editing, review and layout again.

A study whose objective was to develop and assess an educational booklet to promote a healthy lifestyle in people with HIV also achieved an acceptable level of satisfaction in appearance assessment^(27)^. Another study also brought scores considered satisfactory when analyzing items related to appearance, indicating that this aspect makes the material more attractive and motivating for reading^(28)^.

### Study limitations

As limitations of this study, it is pointed out the absence of medical professionals and physical educators in booklet validity, due to non-acceptance of the invitation, even after several contact attempts, bearing in mind that these professionals are also part of the care routine for people living with DM. It is recommended that validity be carried out by the target audience. Furthermore, it is suggested that, in further studies, the effectiveness of this tool can be tested, based on intervention research, either through experiments or quasi-experiments, thus proving the effectiveness of its applicability.

### Contributions to nursing, health or public policy

The educational technology validated in this study becomes innovative due to the theme presented and the target audience, given the complexity of care for people with DM in the face of the COVID-19 pandemic scenario. The importance of this research for developing new resources and strategies for educational practices is highlighted. Thus, the development of this investigation will contribute to the social transformation of people living with DM, in addition to providing an answer to existing knowledge gap on the subject.

## CONCLUSIONS

The objective proposed by the study was achieved through content and appearance validity by experts. The CVR results confirm that the booklet achieved adequate content and appearance validity rates. It is expected that this educational technology will be used to promote the health of people with DM, making them reflect on their current lifestyle and start to adopt measures indicated in the material.

Therefore, it was concluded that the booklet, by adapting to experts’ suggestions and comments, becomes an accurate tool to be used as a health promotion measure for people with DM in the face of COVID-19.
